# Do the People Need Protection from Quacks?

**Published:** 1895-06

**Authors:** 


### DO THE PEOPLE NEED PROTECTION FROM QUACKS?

In an editorial on the subject of the Legislature and the Doctors, in our March issue, we stated, among other things, that it should be explained to the members of the legislature that the reason why the physicians of Texas are asking for the passage of a law that will protect the people from the dangers of the ignorant practitioners of medicine, and from frauds who pretend to heal without medicine is, that the people themselves are really not aware of the danger; they have not the slightest conception of it; they do not know that they are being deceived; that they are in the hands of a quack—in one case, and a fraud in another—for these worthies are shrewd enough to make the majority of their dupes believe that all others are quacks.

The medical practitioners on the other hand, have a very extensive knowledge of these practices, and the evils resulting from them. They know, better than any other class, the extent and nature of the danger incurred by a family who trust an uneducated man, in ignorance of his deficiency, and it is a feeling of humanity which prompts them to ask the strong arm of the law to interpose for their protection. The credulous, the confiding can never be made to see and understand this danger. A medical man can not go to a family who are employing a quack or a faith healer and point out the danger. The reason why, ought to readily suggest itself to even the most thoughtless.

Nor is the danger confined to the ignorant. In every community there are families of prominence, socially, men of liberal education and good intelligence who are afflicted with a hankering after the pretenders, as we know them to be; struck with the Christian Science fad, or who have faith in the magnetic rubbers; and in some communities homeopathy is affected, as a kind of fashion. These deluded persons can all cite you to "cases which have been cured by these means after all the doctors had failed," and nothing can shake their faith; not even the loss of one of their own children.

This latter assertion has been recently very forcibly illustrated by an occurrence at Dayton, Ohio. The child of "Colonel Meade" (*Lancet-Clinic*) whom we take, from the account given, to be a man of intelligence and, from his title, to be above the herd of no-bodies-in-particular, died of tubercular meningitis; died after suffering days and nights of great agony. The family placed the child in the hands of "the Hattons"—professional Christian Science healers—man and wife, and not a physician saw her, nor was a drug, or medicine, or a palliative of any kind administered. Prayer, constant and solely, was the dependence. The death of the child was a crime of omission, and the parents and the healers are the criminals.

At the autopsy the child's father testified his faith in the treatment. He said:

"Hatton is not a physician, but is a Christian Scientist. He does not heal and cure by any method. I sent for him as a Christian Science healer, and because I knew that through his ministrations others had been healed. He gave my daughter what is known as the treatment, using his knowledge of God's law to bring the child in subjection of those laws. Christian Science treatment is reduced to prayer and prayer only. I did not send for a physician because I'd just as soon think of giving my little child poison as the morphine that would have been prescribed, and feeling that way, and having faith in Christian Science that has been tried all over the country."

Poor fellow! Who does not pity him? Who does not feel the same consideration for him we feel for the poor lunatic who thinks he knows it all, and yet is known to be insane? This man is incapable of realizing the danger of trusting such people; ought he not to have the protection of the law? If one sees a blind person about to run into danger unconsciously, is it not ones duty to snatch him away? Yet—according to the claim that is made—this man unquestionably had the right to select the mode of treatment. The difficulty is, they do not know how to select with discrimination.

